# Mode-selective vibrational modulation of charge transport in organic electronic devices

**DOI:** 10.1038/ncomms8880

**Published:** 2015-08-06

**Authors:** Artem A. Bakulin, Robert Lovrincic, Xi Yu, Oleg Selig, Huib J. Bakker, Yves L. A. Rezus, Pabitra K. Nayak, Alexandr Fonari, Veaceslav Coropceanu, Jean-Luc Brédas, David Cahen

**Affiliations:** 1 FOM Institute AMOLF, Science Park 104, 1098 XG Amsterdam, The Netherlands; 2 Cavendish Laboratory, University of Cambridge, JJ Thomson Avenue, Cambridge CB3OHE, UK; 3 Department of Materials and Interfaces, Weizmann Institute of Science, Rehovot 76100, Israel; 4 IHF, TU Braunschweig, and Innovationlab, Speyerer Strasse 4, 69115 Heidelberg, Germany; 5 School of Chemistry and Biochemistry, and Center for Organic Photonics and Electronics, Georgia Institute of Technology, 901 Atlantic Drive NW, Atlanta, Georgia 30332-0400, USA; 6 Solar & Photovoltaics Engineering Research Center, King Abdullah University of Science and Technology, Thuwal 23955-6900, Kingdom of Saudi Arabia

## Abstract

The soft character of organic materials leads to strong coupling between molecular, nuclear and electronic dynamics. This coupling opens the way to influence charge transport in organic electronic devices by exciting molecular vibrational motions. However, despite encouraging theoretical predictions, experimental realization of such approach has remained elusive. Here we demonstrate experimentally that photoconductivity in a model organic optoelectronic device can be modulated by the selective excitation of molecular vibrations. Using an ultrafast infrared laser source to create a coherent superposition of vibrational motions in a pentacene/C_60_ photoresistor, we observe that excitation of certain modes in the 1,500–1,700 cm^−1^ region leads to photocurrent enhancement. Excited vibrations affect predominantly trapped carriers. The effect depends on the nature of the vibration and its mode-specific character can be well described by the vibrational modulation of intermolecular electronic couplings. This presents a new tool for studying electron–phonon coupling and charge dynamics in (bio)molecular materials.

## Abstract (web summary)

The electronic properties of organic molecules are sensitive to structural dynamics, but device control through this phenomenon has not been attained. Bakulin *et al*. show that the photoconductivity can be modulated by selective excitation of molecular vibrations in an organic optoelectronic device.

## Graphical abstract

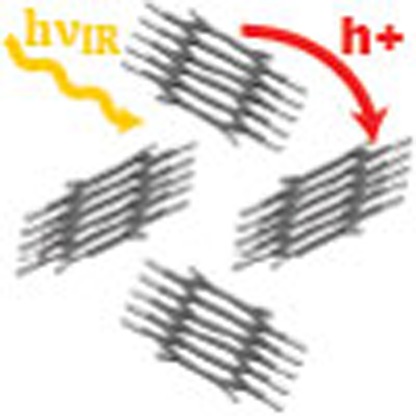

The soft character of organic materials strongly influences their electronic functionality^1^,^2^. In these systems charge hopping and electronic delocalization are determined by the overlap of the molecular orbitals and, therefore, is highly sensitive to minor changes in molecular geometry. Hence, the electronic properties of organic materials are largely determined by the interplay between the electronic and nuclear dynamics of the molecules, referred to as vibronic coupling phenomena. A growing number of interdisciplinary studies show that vibronic effects lie at the heart of a diverse class of effects in physics, chemistry and biology—from nonlinear behaviour of molecular junctions^2^ to photophysics of vision^3^, conformational reorganization^4^ and even olfactory reception^5^. Vibrational motions have been postulated to regulate the interaction between different molecular electronic states by modulating inter- and intra-molecular couplings, by donating or accepting extra energy quanta^5^,^6^, and by suppressing^7^ or promoting^8^ quantum interference phenomena.

Vibronic effects were also shown to be fundamentally important for the conductivity of organic materials. Vibrational motions influence intermolecular electron tunnelling probabilities^9^,^10^,^11^ and govern a variety of non-equilibrium phenomena such as local heating^12^, switching^2^, hysteresis and electronic decoherence^7^,^13^. This makes vibrational excitation a promising tool for spectroscopy of molecular junctions^12^,^14^, tracking charge transfer processes in organic and bio-electronic systems, and, more generally, for the development of electronic devices. For example, remarkable opportunities for organic electronics would arise from the possibility to control charge transport, and, thus, affect device performance by coherently driving nuclear motions along a pre-selected reaction coordinate trajectory. However, despite many encouraging theoretical predictions^15^,^16^,^17^, the experimental realization of vibrationally driven electronics is still elusive due to the complexity of selective control of nuclear motions in an actual electronic junction.

Until now, vibration-associated charge dynamics in organic electronic devices has been only engaged with approaches that do not include mode selectivity. For example, the density and the equilibrium population of vibrational states have been varied via chemical synthesis of molecules with different bond structures^13^ and via thermal population of low-frequency vibrations^7^. However, in principle, it should be possible to access particular non-equilibrium nuclear or vibronic states by using instrumentation of optical time-resolved techniques, such as visible pump–probe^3^,^6^,^18^, time-resolved stimulated/impulsive Raman^19^,^20^ or transient infrared absorption^21^. For example, for inorganic perovskite materials, molecular Mott insulators^22^ and organometallic donor–bridge–acceptor systems^23^,^24^ it has been reported that selective infrared excitation can lead to strong modulation of the electronic properties. Sophisticated all-optical two-dimensional photon echo techniques are even capable of guiding a molecular system through a desired quantum superposition of vibronic/vibrational states^8^,^25^,^26^,^27^,^28^. Although such spectroscopic methods provide a comprehensive approach for probing and controlling molecular motions, and have been applied to model systems such as molecular thin films or solutions, they have not yet been employed to influence charge transport in functional electronic (nano)devices.

In this work, we combine device characterization and ultrafast spectroscopy methods to experimentally demonstrate that the performance of an organic optoelectronic system can be modulated by selectively exciting vibrational modes of the molecules involved in charge transport. As model system we use pentacene/C_60_ bilayer photoresistors. Our experimental approach is based on the interferometric extension of the pump–push photocurrent (PPP) technique. In this work, we extend the PPP method, using the recent progress in ultrafast interferometry^28^,^29^ that allows for a precise control over the time/frequency-domain structure of the infrared optical pulses. We apply a sequence of ultrafast mid-infrared laser pulses to create a coherent superposition of molecular vibrational motions inside the active layer of a device and correlate this excitation with the device performance.

## Results

### Optoelectronic characterization of model device

**Figure 1:**
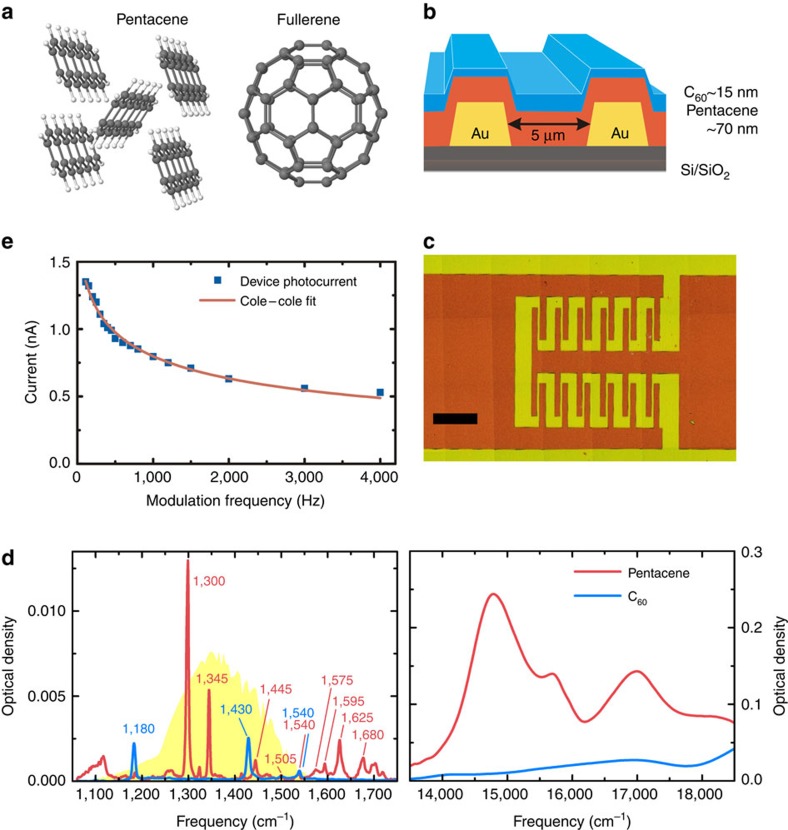
The molecular electronic device characterization. (**a**) Molecular arrangement in the pentacene crystal and C_60_ fullerene structure. (**b**,**c**) Layout and microscope image of the device. The scale bar length is 0.2mm. (**d**) Infrared absorption in the vibrational fingerprint region and optical absorption spectra of pentacene and C_60_. The yellow shaded contour shows a typical laser spectrum used for infrared push. (**e**) Photocurrent from the device as a function of visible light-modulation frequency; the line is a Cole–Cole fit with a 2.2-ms lifetime constant and *α*=0.55 dispersion parameter.

Figure 1a–c describes the organic bilayer photoresistor model system. The active layer of the device consists of polycrystalline pentacene (70 nm) and fullerene C_60_ (15 nm) films (Fig. 1a, Supplementary Fig. 1), thermally evaporated on top of 3-, 5- or 10-μm spaced electrodes arranged in a comb-like geometry on a SiO_2_ substrate (Fig. 1b). We chose this geometry rather than a sandwich-like structure, typical for photodiodes or solar cells, to improve the access of mid-infrared pump pulses to the active layer. Adding the C_60_ layer was critical to enhance the photocarrier generation in the film^30^.

Figure 1d compares the absorption spectra of pentacene and C_60_ in the infrared vibrational fingerprint region and in the region of the optical electronic transitions. C_60_ shows several distinct vibrational modes at 1,180, 1,430 and 1,540 cm^−1^ and has a comparably low optical density in the visible. Pentacene has a rich spectrum of vibrational lines in the infrared and also shows strong excitonic absorption features at frequencies above 14,500 cm^−1^ (690 nm). According to the density functional theory calculations, the strong infrared peaks at 1,300 and 1,345 cm^−1^ are mostly associated with C=C stretching vibrations along the short axis of pentacene, while the weaker high-frequency vibrations correspond to atomic motions mostly aligned with the long axis of the molecule (see Supplementary Fig. 2).

The dark *I*–*V* curves of the devices are symmetric and roughly linear, indicating good hole injection from the gold electrodes to the pentacene layer (see Supplementary Fig. 3). On exposure to visible light, the current flow through the devices strongly increases (approximately three times under 10 mW cm^−2^ illumination). Devices without a C_60_ layer demonstrated only negligible photoconductivity, which indicates that singlet (and triplet)^30^ excitons generated after pentacene excitation are dissociating at the pentacene/C_60_ interface and that the charge generation proceeds through the interfacial charge transfer states^31^. Owing to the large electron injection barrier at the pentacene/Au interface, the dark current is mostly provided by holes, while under illumination both holes and electrons contribute to the photocurrent. Unlike in a typical solar cell, both electrodes are placed below the pentacene films. Therefore, electrons and holes have to pass through the pentacene, which is known to lead to extremely long (up to seconds) extraction times of electrons residing in low-lying trap states in pentacene^32^. This notion is confirmed by the dependence of the photocurrent on the light-modulation frequency (Fig. 1e). Cole–Cole analysis of this dependence shows a typical time constant >2 ms, which we interpret as the lifetime of long-lived electronic charge carriers.

### Pump–push photocurrent measurements

In a PPP experiment, an optoelectronic device is illuminated by a sequence of laser pulses interacting with the active material in the device. The result of these interactions is detected by observing the variations in the current flow through the device as a function of time delay *T* between the pump and push pulses and their spectra. Thus, PPP combines the sensitivity and device relevance of electronic methods with the excitation selectivity and ultrafast time resolution of optical methods. Since its introduction^33^,^34^, PPP has been applied and discussed in the context of photovoltaics^35^,^36^, nanoelectronics, spectroscopy^37^, microscopy^38^ and molecular junction research^14^.

**Figure 2:**
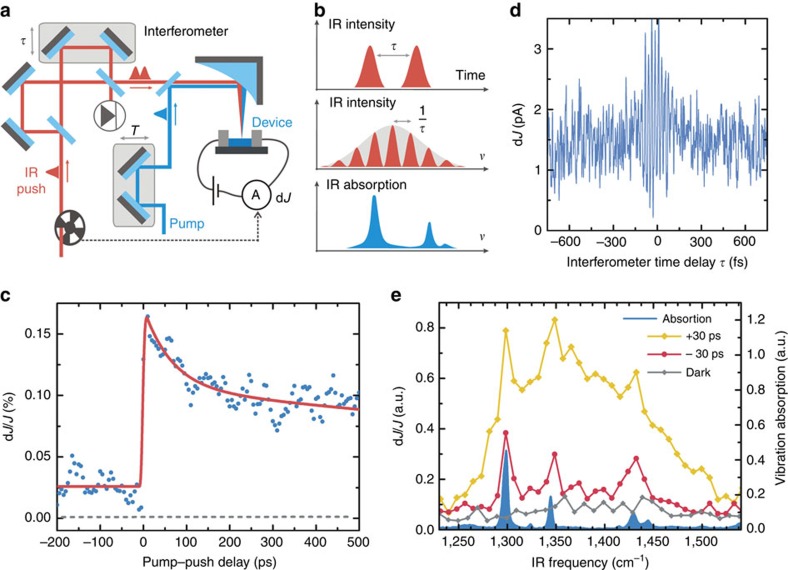
The results of pump–push photocurrent measurements. (**a**) The layout of the time- and frequency-resolved PPP experiment. (**b**) Time- and frequency-domain representations of the infrared (IR) interferometric pulse pair, matching molecular vibrations. (**c**) Broadband PPP transient for pentacene/C_60_ photoresistor. (**d**) Typical PPP interferogram. (**e**) Frequency-resolved PPP signals for pentacene/C_60_ photoresistor, measured as a Fourier transformations of the PPP interferogramms in (**d**) at different pump–push delay times *T*, and a no-pump ‘dark’ measurement.

Figure 2a shows the layout of the experiment, designed to observe the effect of molecular vibrations on the charge transport through the device. The setup combines a 1 kHz visible–infrared ultrafast spectrometer and a lock-in current probe station wired to the device under ∼5 V external bias. First, a visible (15,000 cm^−1^; 665 nm; 1.9 eV) pump pulse illuminates the device. The absorption of the pump light in pentacene leads to the build-up of excitons and charge carriers in the active layer. The generated carriers produce a sequence of 1-ms-spaced current pulses in the measurement circuit with an average photocurrent *J∼*10 nA, detected by the lock-in amplifier at 1 kHz. We note that at such low current densities a charge-induced phase transition^39^ can be excluded. The device is irradiated with a push pulse at certain delay times *T* before or after the pump pulse. The push pulse can promote <1% of the molecules to the excited vibrational state and can also excite low-frequency charge-associated infrared electronic transitions^40^. The effect of infrared light on the charge separation and transport was detected via the variation of device photocurrent d*J*.

Figure 2c presents a typical PPP transient, measured with a single-pulse push (one interferometer arm blocked) at 1,250–1,500 cm^−1^. When the pump was blocked we observed no signal due to the push only. At negative delay time *T*, when the push pulse arrives before the pump, we already observe a substantial increase of the current due to infrared excitation (that is, d*J*>0). We associate this response with the excitation of long-lived photocarriers that were generated by the preceding pump pulse that arrives ∼1 ms earlier. This observation is in line with the long collection times of trapped carriers observed for electrons in pentacene^36^. At delay time *T*=0, the PPP response promptly increases as the concentration of charges in the cell rises due to the arrival of the new pump pulse and the infrared push influences their dynamics. The rapid rise is followed by an ∼100 ps decay component that we assign to the geminate recombination of newly generated charge pairs, which are likely to form electrostatically bound charge-transfer excitons^35^.

In a broadband experiment using a single-pulse push, it is not possible to distinguish the effects of low-frequency electronic excitations from the vibronic phenomena associated with the interference between the molecular vibrational motions and charge dynamics. To separate and address these phenomena individually, we performed push frequency-resolved measurements by exploiting the ultrafast interferometry approach^28^. Using a Mach–Zehnder scheme (Fig. 2a), the push beam is split into two pulses displaced in time by an interferometric delay *τ*. This leads to the formation of a 1*/τ* periodic modulation in the total push spectrum (Fig. 2b), which allows for selective excitation of different coherent superpositions of modes within the bandwidth of the infrared light. In a typical experiment, for a certain pump–push delay *T*, the signal d*J/J* is detected as a function of interferometric delay *τ* (Fig. 2d). The obtained interferogram is Fourier-transformed along the *τ* axis to yield the action spectrum of the push effect.

Figure 2e shows a typical frequency-resolved PPP response of a pentacene/C_60_ device at negative and positive pump–push delay times *T*, and with no pump (dark). At both delays the response consists of a number of narrow peaks on top of a broad featureless response, roughly following the infrared source spectrum. We associate the broad feature with intraband electronic and polaronic absorption, which typically spreads between 1,000 and 5,000 cm^−1^ (ref. 40). The intraband excitation brings the associated charge carriers to a higher-lying delocalized state, thereby enhancing their mobility, decreasing their recombination and thus increasing the current output^35^. The narrow features in the PPP signal match well with the absorption peaks of the vibrational modes of pentacene and C_60_. Therefore, these features in the frequency-resolved PPP response are assigned to the excitation of molecular vibrations that modulate the electronic dynamics. Interestingly, the broad electronic response dominates the PPP signal when the push arrives after the pump, while the vibrational features have similar amplitudes (within the experimental accuracy) at positive and negative *T* delays. This observation indicates that the infrared electronic excitation substantially promotes charge separation at the pentacene/C_60_ interface soon after exciton generation. At the same time, the effect of vibrational excitation is present for long-lived trapped charge carriers and, therefore, does not influence charge separation, but only carrier de-trapping dynamics^35^.

We now focus on the analysis of the vibrational features only. The effect of broadband electronic infrared excitation on charge dynamics in organic semiconductors has been investigated previously^35^, and is outside the scope of this paper. To study the effect of vibrational excitation for a broader set of vibrational modes, we use a wide push spectral window of 1,150–1,700 cm^−1^ and long *τ*-scanning to obtain high-frequency resolution. We also applied time-domain filtering (see Supplementary Fig. 4 and Supplementary Note 1) to suppress broad features due to electronic excitation and nonlinear field-induced tunnelling currents, and performed measurements at using different push spectra (Supplementary Fig. 5).

**Figure 3:**
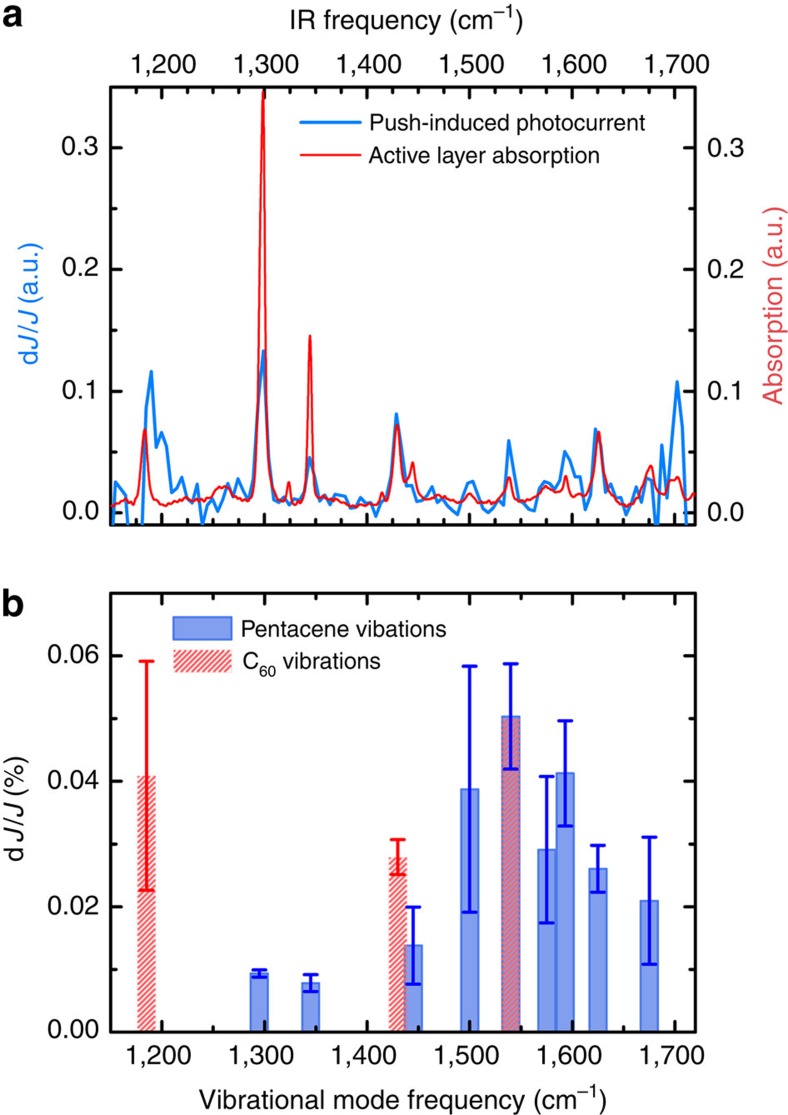
Experimental evaluation of vibrational modulation effect. (**a**) The vibrational part of the PPP response, measured at negative delay time (−100 ps). The signal amplitude is normalized to the spectral density of the infrared (IR) push source. The spectrum is obtained using two PPP spectra, each covering a different but overlapping wavenumber range; these are scaled to match the amplitude of the 1,430 cm^−1^ mode that is present in both spectra. For comparison, the absorption spectrum of the pentacene/C_60_ layer is presented in red. (**b**) The influence of different vibrations on device photocurrent, estimated by normalizing the amplitude of the PPP signal to the absorbed infrared intensity. The change of photocurrent absolute value corresponds to a flat 0.5 μJ cm^−2^ per cm^−1^ spectral density of exciting infrared light, fully absorbed by the vibrations. The error bars (red for fullerene modes, blue for pentacene vibrations) are s.d. for measurements on different devices; the number of measurements was 10 for 1,300–1,450 cm^−1^ modes and 4 for all other modes.

Figure 3a presents the vibration-associated PPP spectrum covering most of the infrared fingerprint frequency range. The amplitude of the PPP response was normalized to the spectral density of the push pulse to allow for a direct comparison of the different vibrational lines. The spectrum is the result of several measurements with different push frequencies spliced together to match the amplitude of the effect for the 1,430 cm^−1^ feature, which was present in all measurements. In the 1,150–1,700 cm^−1^ region, we observe 12 PPP peaks at frequencies that match well the infrared-active vibrational modes of pentacene and C_60_.

We note that, while the vibrations of charged pentacene may differ from those of the neutral molecules^41^, these differences should not be observed in the PPP data. First, the shift in frequency for most individual modes is small^42^,^43^ and, for most modes, below our frequency resolution (∼10 cm^−1^). This conclusion is also supported by density functional theory (DFT) calculations, see Supplementary Tables 1 and 2. Second, the minor shifts of the vibrational levels lead to a highly efficient vibrational energy transfer^44^ between neutral and charged molecules, which allows the vibrational excitation of neutral pentacene to be delivered to the charge-trapping sites. Third, according to Miller–Abrahams (MA) formalism, when a carrier hops from a radical to a neutral state all vibrational modes coupled to these electronic states contribute to the transfer rate^45^. Therefore, it is not surprising that the lattice vibrations, that is, those of the neutral pentacene, are observed in the de-trapping dynamics. We note that this mechanism does not contradict the mode-selective nature of the vibronic coupling.

## Discussion

We observe that the amplitude of the PPP response does not follow the intensity of the infrared absorption. For example, the band at 1,345 cm^−1^ possesses a much stronger infrared absorption than the 1,630 cm^−1^ vibration, but shows a weaker PPP response. This result shows that the observed PPP response cannot be explained by the equilibration of vibrational energy between modes and average heating of the device active layer, thus illustrating the mode-selective character of the PPP response. This example illustrates that different atomic motions couple differently to the charge dynamics of the system. To exclude that the non-scaling of the PPP response with infrared absorption is merely an effect of a different orientation of the vibration dipoles with respect to the exciting infrared light, we also performed angle-dependent infrared absorption measurements. These measurements showed that the modes exhibiting very different PPP effect, for example, at 1,345 and 1,630 cm^−1^, have similar dipole orientations (see Supplementary Fig. 6), which rules out orientation effects.

Figure 3b compares the effect of vibrational excitation on the device photoconductivity for different vibrational modes, obtained by normalizing the PPP response to the number of photons absorbed by the vibrational mode. In accordance with Fig. 3a, the 1,300 and 1,345 cm^−1^ modes show the weakest coupling. The higher-frequency vibrations of pentacene show a 5–8 times higher effect on the photoconductivity. For two of the fullerene vibrations the effect is similar to that of the high-frequency vibrational modes of pentacene.

These results can be rationalized in the framework of the phonon-assisted MA theory^45^. According to this model, carrier hopping from a trapping state to higher-energy (more conducting) states takes place via absorption of a phonon with energy to compensate for the energy difference between initial and final electronic states; the hopping rate *k* is defined by the electron–vibrational coupling constant (*v*) and the occupation number (*n*_Δ_) of the absorbed phonon, that is, *k∼v*^*2*^*n*_Δ_. In thermal equilibrium, the occupation number of a high-energy molecular vibration is very small, *n*_Δ_=exp(−Δ/*k*_B_*T*). For a comprehensive description of the PPP response the interaction with the infrared photons should be included into the MA model. However, at the conceptual level the effect can be understood by assuming that an infrared excitation creates a non-equilibrium population of the molecular vibrational manifold; therefore, an increase in the hopping probability is expected. The mode-selective character of the PPP response is therefore defined by the electron–vibration coupling constants.

To achieve mechanistic insight into the observed phenomena, we performed a theoretical analysis of the coupling between the different molecular vibrations and the charge carriers (holes) in pentacene. In molecular systems these couplings can be divided into two types, that is, local (Holstein type) and non-local (Peierls type)^46^. The Holstein electron–phonon interaction originates from the modulation of the site energies by the vibrations. Only totally symmetric molecular vibration modes can contribute to this interaction. For centrosymmetric molecules like pentacene, the symmetric modes are not infrared active. While charge-induced symmetry breaking can lead to the infrared activation of Raman modes in polymers, such an effect has not been observed and is most likely negligible for pentacene. The Peierls-type electron–phonon couplings are associated with the dependence of the transfer integrals on the distances between adjacent molecules and their relative orientations^46^. For this type of coupling, there are no symmetry restrictions.

**Figure 4:**
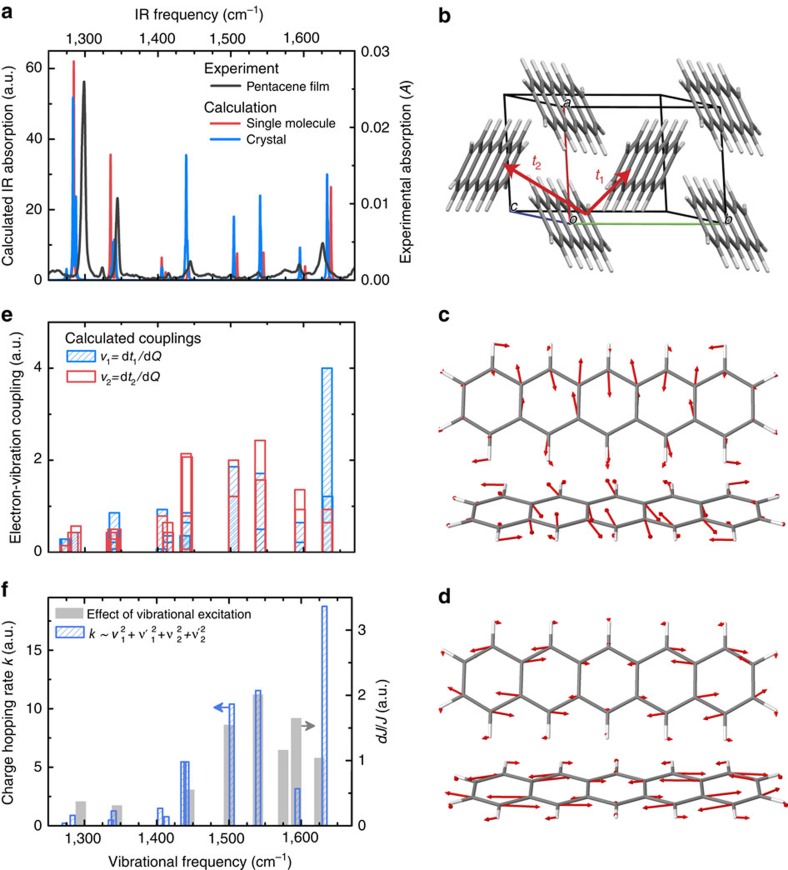
Calculated electron–phonon couplings and comparison with experiments. (**a**) Simulated infrared (IR) spectrum for the pentacene molecule (red) and crystal (blue), superimposed on the experimental spectrum for comparison. (**b**) Molecular crystal structure and transfer integrals *t*_*i*_ addressed in the calculations. (**c**,**d**) Eigendisplacements for the modes with large transition dipoles at 1,288 cm^−1^ and 1,632 cm^−1^. (**e**) Non-local electron–phonon coupling constants for two largest transfer integrals. (**f**) Calculated vibration-induced hopping rates for the different modes that were addressed experimentally, compared with the experimentally observed effect of vibrational excitation on the photocurrent, d*J*/*J*.

On the basis of previous studies^47^, we used a triclinic polymorph^48^ to represent the pentacene layer structure in the calculations. Figure 4a shows the simulated infrared spectra for a single pentacene molecule and for the crystal in comparison to the experimental infrared absorption. The agreement between the experimental and calculated vibrational frequencies (with typical discrepancies <10 cm^−1^) allows the assignment of the vibrational modes observed in the frequency-resolved PPP experiment. The 1,300 and 1,345 cm^−1^ features in the experimental spectrum are associated (Fig. 4c and Supplementary Figure 2) with in-plane ring stretching modes along the short axis of pentacene^49^,^50^, while the infrared peaks at 1,540 and 1,630 cm^−1^ are associated with molecular deformations along the long axis of pentacene (Fig. 4d and Supplementary Figure 2).

The non-local hole-vibration couplings are defined as the derivatives of the charge-transfer integrals with respect to the vibrational coordinates, *v*_*j,i*_*=*d*t*_*i*_*/*d*Q*_*j*_, and can be computed numerically^15^,^51^. Both the transfer integrals and electron–vibration couplings have been derived in a one-electron approximation (see Methods for details). Our results indicate that there are two main transfer integrals contributing to charge transfer in the pentacene crystal: *t*_1_*=*75 meV and *t*_2_*=*32 meV; both are associated with intermolecular interactions along the herringbone directions (see the red arrows in Fig. 4b). The other two transfer integrals oriented along the *a* axis are substantially smaller and do not demonstrate substantial modulation by infrared-active modes (see Supplementary Fig. 1). The derived coupling constants of the infrared-active modes are shown in Fig. 4e. The couplings in the 1,400–1,650 cm^−1^ range are about 2–5 times larger than in the 1,200–1,400 cm^−1^ range. To link the variations in coupling and the probability to de-trap a charge with a vibrational excitation, we estimated the rates of vibration-induced charge hopping. In the case of two pathways, the rate is defined within perturbation theory as: 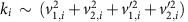, where *v*_*j,i*_ and 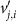
correspond to quasi-degenerate molecular vibrations of similar frequencies. Figure 4f presents these hopping rates together with the experimental observations from Fig. 3b. The theoretical results are consistent with the experimental data, the theory/experiment correlation coefficient being 0.72 in 1,280–1,600 cm^−1^ region (Supplementary Fig. 7). In particular, calculations capture well the mode-selective character of the phenomena, with the modes below 1,430 cm^−1^ calculated to have a much smaller impact on charge hopping than the higher-frequency ones. On the basis of the calculations, the intermolecular electronic couplings and charge transport in pentacene crystals are seen to be most sensitive to stretching deformations along the long molecular axis, while the stretching deformations along the short molecular axis are less important.

In conclusion, we demonstrated that the vibrational coupling phenomena, which play an essential role in molecular-scale charge transport, can be explored and put to action by combining optical and electronic techniques. Both the experiment and theoretical calculations demonstrate that different non-equilibrium geometries and atomic motions have different effects on the charge dynamics. Specifically, our results show that vibrations along the long axis of pentacene molecules lead to a stronger increase of hopping transport via charge de-trapping than vibrations along the short axis. The mode-selective vibrational approach to influence charge dynamics introduced here opens up a plethora of opportunities for basic research, including the development of high-mobility organic semiconductors, and the utilization of vibronic phenomena for ultrafast switching of organic devices. In addition, the mode-selective and local nature of our method might be particularly useful for the identification of charge transport mechanisms and pathways in (bio)molecular junctions^52^.

## Methods

### Materials and devices

The gold electrodes for the phototransistor were fabricated on a SiO_2_ substrate by a standard microfabrication technique with sequential processes of photolithography, metal evaporation and lift-off. Subsequently, 70 nm pentacene and 15 nm C_60_ films were thermally evaporated without substrate heating at a rate around 0.5 Å s^−1^. Samples were kept in N_2_ atmosphere from fabrication until the ultrafast measurements. *IV* curves were measured using a commercial source-measurement unit (Agilent B2900A) and a halogen lamp for light/dark measurements.

### Ultrafast experiments

Ultrafast experiments were performed inside a N_2_ flow compartment. Pump and push pulses were generated by using the output of a 1-kHz repetition rate femtosecond regenerative amplifier (800 nm, 35 fs, 4 mJ per pulse), which by employing a beam splitter (3:1) pumped a pair of optical parametric amplifiers (TOPAS). To generate the pump pulse, the ‘signal’ output of one of the TOPAS was frequency doubled in beta barium borate (BBO) crystal. For infrared-push generation the signal and idler pulses from the other TOPAS were difference-frequency mixed in a AgGaS_2_ crystal. This provided a femtosecond (100 fs, ∼20 μJ) infrared pulse centred at 1,360 or 1,550 cm^−1^. After this infrared light entered a Mach–Zehnder interferometer a pair of collinear push pulses was produced with the spectrum^28^: *I*_pp_(*ω*)=*I*(*ω*)(2+2cos(*ωτ*)) where *I*(*ω*) is the spectrum of a single push pulse; *τ* is the delay between the two pulses. The interferometer was controlled using a reference beam from a He–Ne laser following the path of the infrared beam. Both pump (<10 nJ) and push (∼1 μJ) pulses were focused on the device using a *R*=15 cm spherical gold mirror. The device was biased (∼5 V) using a battery voltage source. Current modulation d*J* was detected using a lock-in amplifier (Stanford Research 830) in the current mode locked to the mechanical modulator (370 Hz) in the push beam. PPP interferogram was recorded as a function of the delay between the two push pulses *τ*. The Fourier transformation of this time variable yielded the pump-frequency axis in the PPP spectrum. All measurements were performed at room temperature and N_2_ flow atmosphere.

### Details of theoretical calculations

Geometry optimizations of the crystal structure of the triclinic pentacene polymorph proposed by Campbell^48^ were performed at the PBE0/6–31G level of theory. During the optimization, the unit-cell parameters were kept fixed at the experimental values. A uniform 8 × 6 × 4 Monkhorst–Pack k-point grid was employed. The Γ-point phonons (lattice vibrations) within the harmonic approximation and the infrared intensities were obtained via a coupled perturbed Hartree–Fock approach. These calculations were carried out with the CRYSTAL14 package^53^,^54^. Since there are two independent molecules in the unit cell of the pentacene crystal, all molecular vibration modes are (quasi)degenerate. All lattice vibrations in the region of interest can be visualized on the Internet (http://afonari.com/pentacene-vibrations/).

Transfer integrals (electronic couplings) for holes were calculated using a fragment orbital approach based on the unperturbed highest occupied molecular orbitals of the individual neutral molecules extracted from the optimized crystal geometry^55^. The non-local hole–phonon coupling constants (*v*) come from the modulation of the transfer integrals by lattice vibrations. The coupling constants can be computed by expanding the electronic couplings into Taylor series of the phonon eigenvectors: 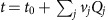. Here, *t*_0_ is the electronic coupling at the crystal equilibrium geometry; *Q*_*j*_ is the normal-mode coordinate of mode *j*; *v*_*j*_ is the linear non-local electron–phonon coupling constant. In practice, the coupling constants are calculated by distorting the crystal along all normal-mode coordinates with positive and negative steps and then computing numerically the related derivatives of the transfer integrals for each vibrational mode.

All the DFT calculations for the isolated neutral and positively charged molecules were performed at the PBE0/6–31G level of theory using the Gaussian package^56^. The normal modes of the neutral (*Q*_n_) and cation (*Q*_c_) states are related to each other via a multidimensional rotation (Duschinsky) matrix (Supplementary Table 2)^57^. The Duschinsky matrix was computed with the DUSHIN code^58^. The calculated frequencies for both isolated molecule and crystal were scaled by 0.95 (Supplementary Table 1)^59^.

## Additional information

**How to cite this article:** Bakulin, A. A. *et al*. Mode-selective vibrational modulation of charge transport in organic electronic devices. *Nat. Commun.* 6:7880 doi: 10.1038/ncomms8880 (2015).

## Supplementary Material

Supplementary Information Supplementary Figures 1-7, Supplementary Tables 1-2, Supplementary Note 1 and Supplementary References
