# Supplementary material for: Mode-selective vibrational modulation of charge transport in organic electronic devices
Source: Nat Commun. 2015 Aug 6;6:7880. doi: 10.1038/ncomms8880 (PMC4538862; doi:10.1038/ncomms8880)
Supplement: Supplementary Information — Supplementary Figures 1-7, Supplementary Tables 1-2, Supplementary Note 1 and Supplementary References [file ncomms8880-s1.pdf]

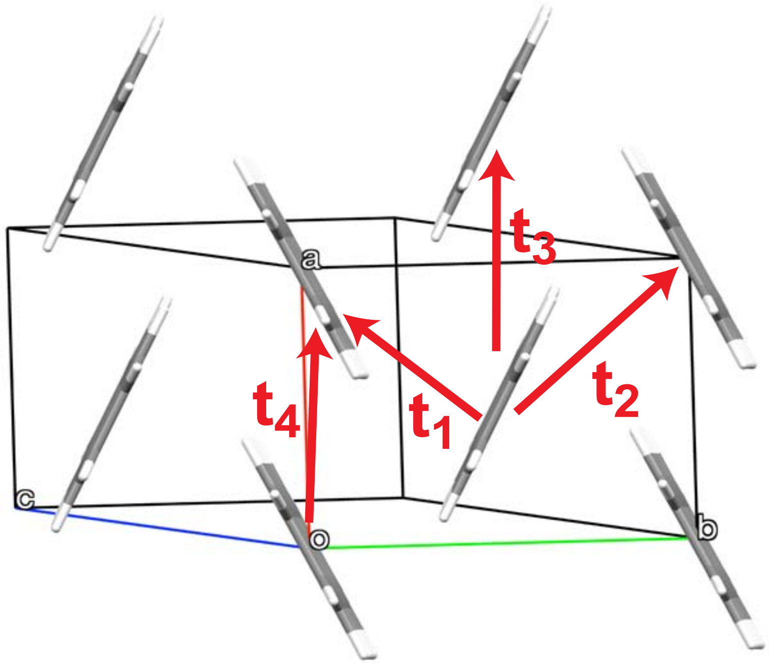

Supplementary Figure 1. Crystal packing of pentacene. The largestholecharge transfer integrals are shown in red:t_1 = 75 meV, t_2 = 32 meV, t_3 = 20 meV, t_4 = 6 meV. Note that IR-active modes do not modulate transfer integrals t_3, t_4.[1]

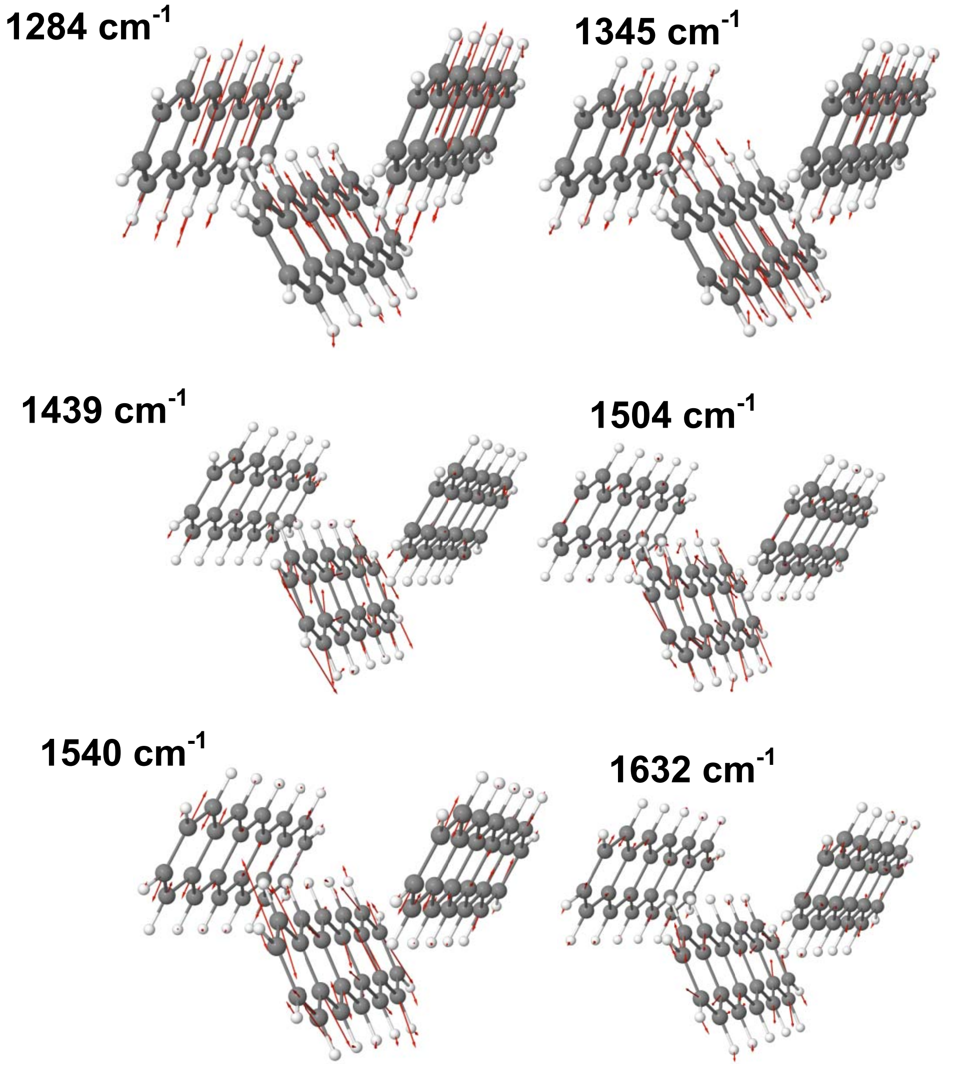

Supplementary Figure2. Vibrational modes. Eigendisplacementscorresponding tothe vibrational modes addressed in the study.

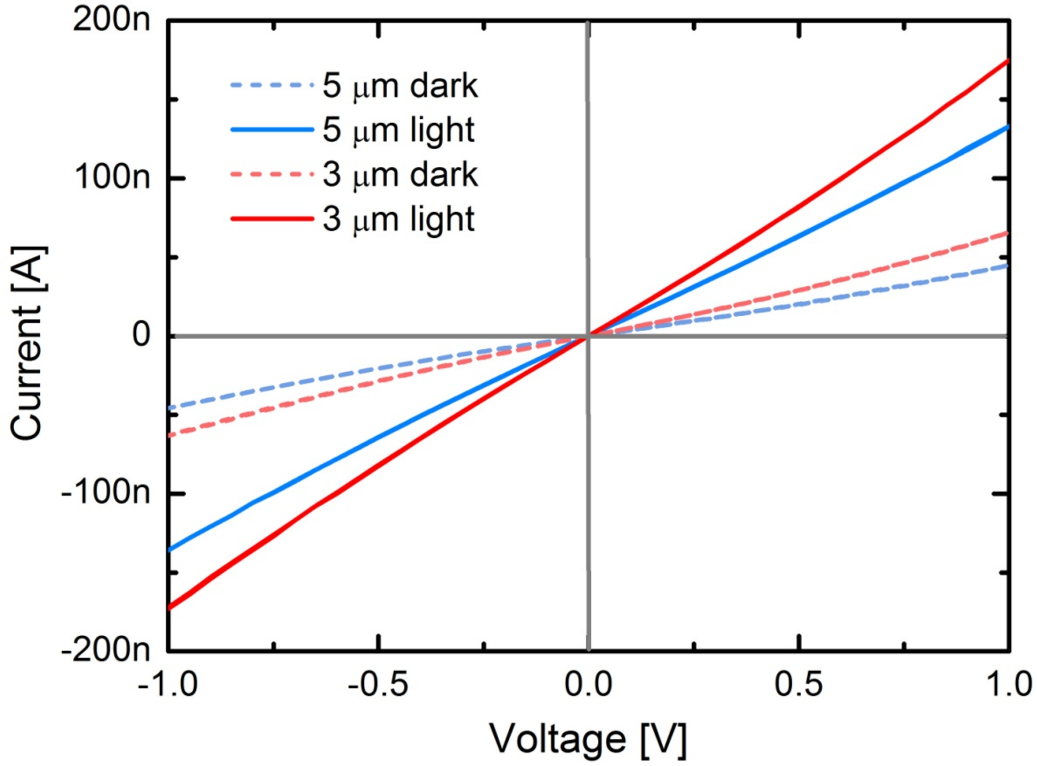

Supplementary Figure 3. Device characterization. Typical I-V characteristics of the studied pentacene/C_{60} devices with 3 and 5 μm electrode spacing; visible-light illumination was about 10mW/cm^2 provided by a halogen lamp.

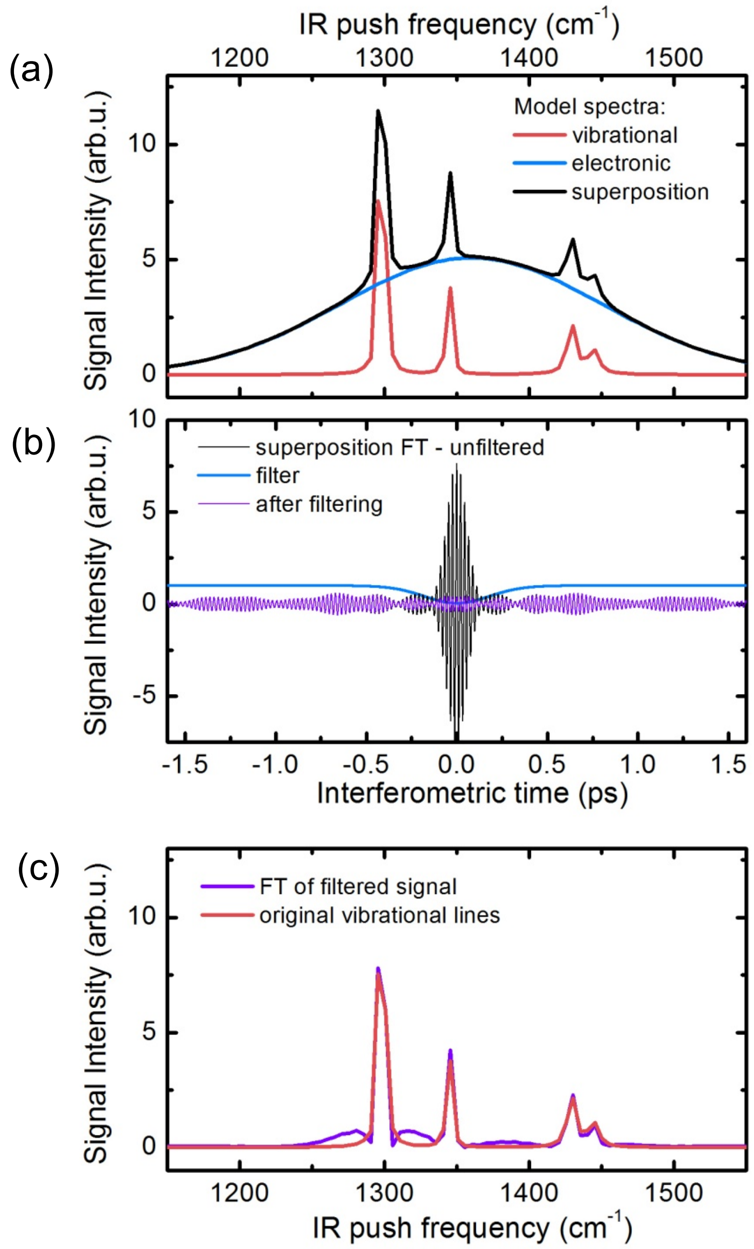

Supplementary Figure 4. Illustration and accuracy check for the time-domain filtering procedure. (a) Red and blue lines show the 'model' dJ/J responses due to push-induced vibrational and electronic transitions. Black curve is a superposition of responses typically observed in the experiments. (b) Black line shows time-domain representation of the superposition curve from figure a; this represents typical experimentally observed interferogram. Blue line shows a Gaussian time-domain filter. Purple line shows a filtered interferogram. (c) Purple line is a PPP spectrum obtained by FT of filtered interferogram. It is almost identical to the vibrational part of the input signal (a).

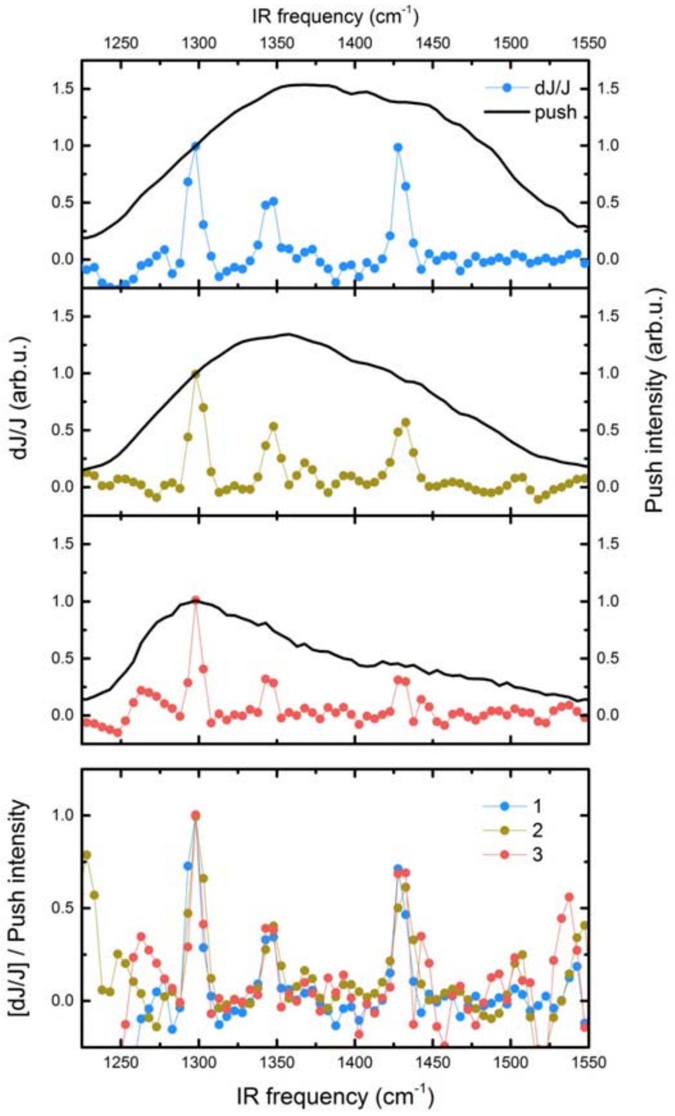

Supplementary Figure 5. Data reproducibility at different experimental conditions. Top three panels present PPP vibrational responses measured for three different devices with different IR-push pulses. The bottom panel shows the relative influence of different modes on photocurrent deduced after the normalisation of PPP responses on the respective push spectra.

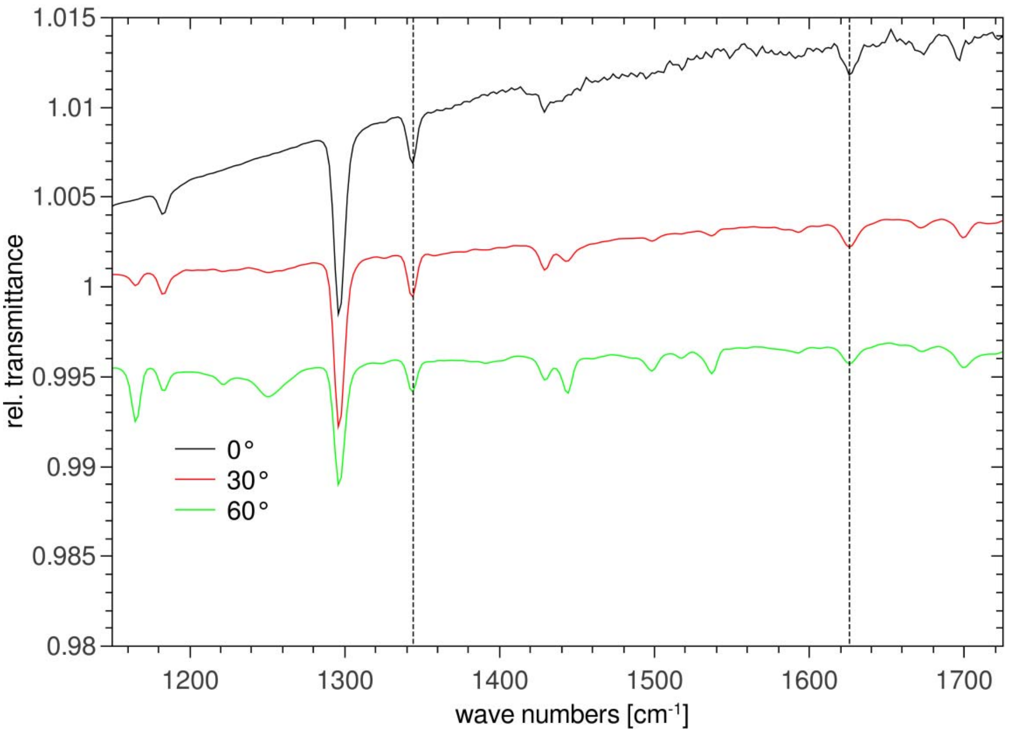

Supplementary Figure 6. Transmission anisotropy of pentacene film. Relative transmittance spectra of a pentacene/C_{60} (70nm/15nm) film on Si at different angles of incidence (with respect to the surface normal). A strong increase in peak height with increasing angle points to a transition with dipole moment perpendicular to the surface, whereas a decrease points to a dipole moment parallel to the surface. The two marked peaks at 1345 cm^{-1} and 1630 cm^{-1} behave similar under tilt (indicating similar orientations for the transition dipoles), but exhibit very different PPP couplings (~3 times stronger for the vibration at 1630 cm^{-1}). We conclude from this observation that the non-scaling of the PPP response with IR absorption is not merely an effect of different orientation of the dipoles, but does reflect the different coupling strengths between charge carriers and vibrations.

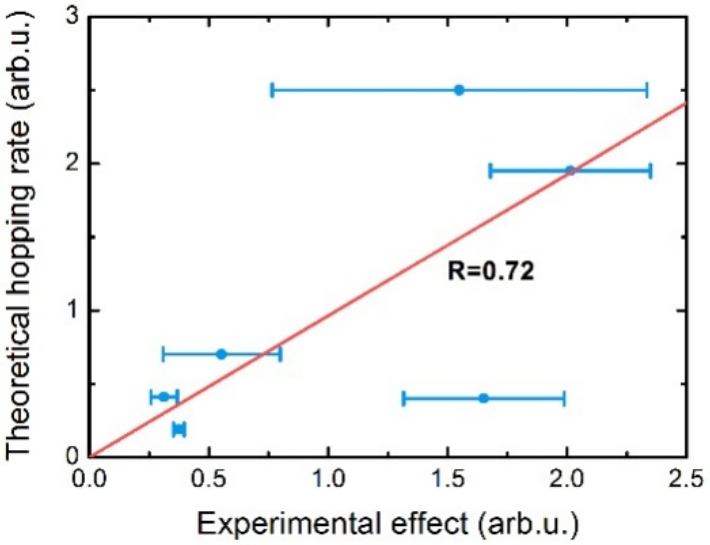

Supplementary Figure 7. Theory vs. Experiment correlation. Correlation analysis of the vibrational effect on charge de-trapping observed in experiment and derived from the calculations. Linear fitting yields the correlation parameter of R=0.3 when all points are included and more convincing R=0.72 when the mode at 1620cm^{-1} is excluded due to proportionally high calculated hopping rate. The correlation value of 0.72 indicates the adequateness of the model taking all the simplifications necessary for the calculations.

Supplementary Table 1. IR intensities and nonlocal hole-phonon couplings for pentacene.

Crystal				Neutral molecule		Charged molecule	
**Freq., cm^{-1}**	**IR, km/mol**	**v_1, meV**	**v_2, meV**	**Freq., cm^{-1}**	**IR, km/mol**	**Freq., cm^{-1}**	**IR, km/mol**
970.13	77.04	0.21	0.07	913.29	129.07	939.34	93.61
970.63	36.75	0.14	0.07	958.03	17.55	977.01	21.64
992.53	21.65	0.43	0.43	995.98	5.69	1013.59	0.09
994.90	0.09	0.79	2.86	1113.81	7.96	1116.32	3.92
997.79	28.24	0.00	0.79	1133.71	4.91	1166.38	28.84
1001.41	2.51	0.14	0.21	1163.09	0.26	1176.69	198.32
1110.80	36.33	0.07	0.00	1186.55	3.62	1188.85	2.75
1113.74	16.09	0.07	0.14	1228.96	1.66	1235.58	131.09
1137.16	4.55	0.29	0.43	1268.43	0.01	1273.24	2.23
1138.36	5.14	0.07	0.93	1284.67	24.38	1288.99	41.72
1158.93	5.68	0.07	0.29	1335.15	13.96	1336.43	6.29
1164.37	36.81	0.07	0.07	1342.13	2.51	1380.13	653.83
1187.31	2.96	0.29	0.07	1405.31	2.50	1400.18	839.40
1190.04	41.10	0.07	0.07	1410.59	0.87	1415.26	148.93
1228.30	14.87	0.29	1.14	1440.56	0.86	1438.87	20.08
1230.19	1.28	0.50	1.00	1442.49	1.27	1444.38	6.94
1272.59	1.01	0.29	0.14	1508.64	2.99	1488.77	502.01
1274.71	4.63	0.29	0.14	1544.89	3.08	1530.31	292.10
1283.62	81.01	0.43	0.43	1601.88	1.56	1578.82	3.89
1287.69	36.16	0.43	0.57	1637.32	10.38	1598.20	20.13
1336.47	4.64	0.07	0.43				
1338.68	15.22	0.21	0.50				
1340.40	15.86	0.86	0.29				
1342.41	16.93	0.43	0.50				
1404.86	4.47	0.07	0.00				
1406.01	4.80	0.93	0.79				
1413.53	0.14	0.21	0.64				
1414.51	0.63	0.36	0.43				
1437.36	9.19	0.36	0.07				
1438.79	21.27	0.86	2.14				
1438.95	33.94	0.36	0.79				
1440.53	12.79	0.64	2.07				
1503.93	6.16	1.21	2.00				
1504.28	23.99	1.86	1.21				
1540.27	34.73	1.71	2.43				
1541.25	14.26	0.50	1.57				
1594.79	12.50	0.64	1.36				
1595.31	3.98	0.21	0.93				
1632.15	43.92	4.00	0.93				

Supplementary Table 2. Correspondence between frequencies of cation and neutral molecule together largest coefficients of Duschinsky matrices.

Cation freq., cm^{-1}	Neutral freq., cm^{-1}	%
939.34	913.29	99.6
977.01	958.03	99.7
1013.59	995.98	99.2
1116.32	1113.81	98.8
1166.38	1133.71	73.8
	1163.09	22.3
1176.69	1133.71	21.5
	1163.09	76.7
1188.85	1186.55	98.1
1235.58	1228.96	96.7
1273.24	1268.43	93.8
1288.99	1284.67	95.6
1336.43	1342.13	96.1
1380.13	1335.15	18.5
	1405.31	52
	1544.89	10.1
1400.18	1335.15	68.5
	1410.59	11.5
1415.26	1405.31	26.6
	1410.59	56.8
1438.87	1410.59	88.6
1444.38	1442.49	97.2
1488.77	1508.64	77.9
1530.31	1544.89	77.8
1578.82	1601.88	88.5
	1637.32	10.1
1598.20	1601.88	10.2
	1637.32	88.9

Supplementary Note 1. Time domain Fourier filtering.

The experimentally detected responsewas typically a superposition ofvibrational response (narrow lines in the frequency domain) as well as electronic and field-induced tunneling responses (broad bands in the frequency domain).[2,3]To extract the vibrational signals from the total response we have developed a time-domain filtering procedure which effectively suppressed all the broad feature in PPP spectra.

First the interferometric delay $\tau=0$is determined using the signal in the reference arm of Mach-Zehnder interferometer. The $dJ/J(\tau)$interferogramis then 'zeroed' - shifted accordingly along the interferometric time axis. After this the interferogram is multiplied by the following filtering function:

$$f(\tau)= 1-[0.95\ exp(-\tau^2/2\Delta\tau^2)]$$

Where, $\Delta\tau$ is a phenomenological filtering parameter, typically comparable to the inversed bandwidth of IR push spectrum ($\Delta\tau$ ~200 fs). Such filtering leads to the suppression of the central part of the interferogram and tothe corresponding exclusion of broad features from the Fourier Transform (FT) PPP spectrum. At the same time, such filtering does not alter the phase information contained in the data which makes it more stable compared to any other filtering procedure we employed.

The robustness of time-domain filtering is illustrated in the figure 5S. A model superposition spectrum of narrow and broad spectral features (Fig. 5Sa) is used to generate a model interferogram containing both 'electronic' and 'vibrational' responses (Fig. 5Sb, black line). After the filter (Fig. 5Sb, blue line) being applied, a new interferogram (Fig. 5Sb, purple line) is produced. This new interferogram contains exclusively vibrational response which is only slightly distorted compared to the input data (Fig. 5Sc).

Supplementary References:

1 Girlando, A. *et al.* Interaction of charge carriers with lattice and molecular phonons in crystalline pentacene. *J Chem Phys* **135** (2011).

2 Bakulin, A. A. *et al.* The Role of Driving Energy and Delocalized States for Charge Separation in Organic Semiconductors. *Science* **335**, 1340-1344, (2012).

3 Schiffrin, A. *et al.* Optical-field-induced current in dielectrics. *Nature* **493**, 70-74, (2013).
